# Core conflictual relationship theme: the reliability of a simplified scoring procedure

**DOI:** 10.1186/s12888-020-02558-4

**Published:** 2020-04-06

**Authors:** Peter Tallberg, Randi Ulberg, Hanne-Sofie Johnsen Dahl, Per Andreas Høglend

**Affiliations:** 1 Division of Mental Health and Addiction, University of Oslo, Oslo, Norway; 2 Department of Psychiatry, Diakonhjemmet Hospital, Diakonveien 12, 0370 Oslo, Norway; 3 Research Unit, Division of Mental Health, Østfold Hospital Trust, PO Box 300, 1714 Grålum, Norway; 4 Research Unit, Division of Mental Health and Addiction, Vestfold Hospital Trust, Box, 2169, 3125 Tønsberg, Norway

**Keywords:** Relationship patterns, Psychodynamic, Core conflictual relationship theme, Rater agreement

## Abstract

**Background:**

Creating a case formulation is an important and basic skill in psychotherapy meant to guide treatment. A patient’s interpersonal pattern is an essential part of a case formulation. Core Conflictual Relationship Theme (CCRT) is a well-known structured method to describe interpersonal patterns.

The CCRT method is based on the assumption that humans display a central relationship theme, which is shown in most relationships as well as in the patient-therapist relation. The CCRT scoring is based on how the patient describes interactions with others, in therapy sessions or in a specific interview. These descriptions are transcribed. Raters then score the identified relational episodes by choosing elements from the clustered categories of Wishes, Response from Others and Response from Self. The method has shown high validity and reliability. Inter rater reliability is generally good: Cohen’s kappa ranging from 0.55 to 0.70.

To decide CCRT pattern from transcribed material is time consuming and labour intensive This study investigates a labour- and timesaving version of the method.

**Methods:**

This study aimed to investigate rater agreement in a simplified method of scoring the CCRT, based directly on live semi-structured dynamic interviews without transcribing the material.

Fifty-two patients referred for psychotherapy in a clinical trial, were scored for CCRT pattern. Based on information that came forth during the two-hour interview, raters scored the patients choosing elements from the clustered categories of Wishes, Response from Others and Response from Self. More than one category in each component could be chosen without ranking. Five raters compared two by two were investigated. Inter rater reliability was measured by Cohen’s kappa.

**Results:**

Mean kappa for Wishes, Response from Others and Response from Self was .33, .41 and .45 respectively. Mean kappa for CCRT in total was .41 among 5 raters.

**Conclusion:**

In this simplified method to score the CCRT based on oral dynamic interviews, fair to moderate IRR was obtained.

**Trial registration:**

First Experimental Study of Transference-interpretations (FEST307/95).

Registration number: ClinicalTrials.gov Identifier: NCT00423462.

## Background

Creating a case formulation is an important and basic clinical skill. Based on the patient’s narrative, it is a tool for the therapist to structure ideas about what has caused the patient’s problems, why the problems persist and how to make the patient feel better [1]. The case formulation can fill the gap between diagnosis and treatment and can be perceived as lying at the intersection of aetiology and description, theory and practice, and science and art [2]. When developed, the case formulation is based on the psychotherapist’s knowledge and preconceptions, what the patient has chosen to reveal, how a patient is presenting himself and interacts with the therapist. In this study a simplified method to describe patients is tested.

The primary function of a case formulation is to guide treatment. A psychodynamic formulation includes motivational components, aims to identify central dynamic patterns that repeat themselves over and across situations resulting in distress or psychological limitations, and guides clinicians in minimizing inferences from observable clinical data [3]. Many therapists have the opinion that case formulation is a time- consuming and difficult process, therefore, a simple mental construction would be sufficient.

Bowden [4] reported that 90% of educators in a study ranked case formulation skills as very important, even essential. However, case formulation skills are difficult to acquire. Kuyken and colleagues [5] studied the quality of case formulations constructed by 115 health professionals, 44% of the formulations were considered good enough. Eells and colleagues [6] evaluated 56 intake formulations, of which less than half described predisposing life events and/or inferred psychological mechanisms, as necessary in a proper case formulation.

Seitz [7] summoned a group of psychoanalysts in an effort to create reliable case formulations but they never succeeded. He concluded that they applied different levels of inference to the clinical data and hence never agreed on what was centrally important. A basic prerequisite in formulating cases is a certain level of agreement among raters. In their review, Barber and Crits-Christoph [8] found that structured case formulations can attain reliability. This is confirmed in a study by Garb [9] with clinicians sharing the same theoretical background. A recent publication also shows that very experienced clinicians with similar theoretical stance produced reliable, and thus clinically relevant formulations without elaborate instructions about how to structure the case formulations [10].

Psychotherapy is interpersonal in nature. Several methods have been developed over the last decades to identify and describe interpersonal patterns in psychotherapy, often as part of a case formulation. The most well-known is the Core Conflictual Relationship Theme (CCRT) method [11].

The CCRT method constructed by Luborsky and Crits-Christoph is based on the assumption that humans display a central relationship theme or transference pattern [12]. That is, people in general display the same patterns with different people, in various relationships, including the patient-therapist relationship [13]. In several studies this method has shown high reliability in identifying peoples wishes, responses from others and responses of self [11, 14]. There is also evidence for the validity of the CCRT [15, 16], [14]. The CCRT has strong convergent validity with other transference- related measures such as the circumplex model of human reactions in relation to others, the Structural Analysis of Social Behavior (SASB) [17, 18].

The CCRT scoring is based on narratives presented by the patient [19]. The narratives should describe interactions with others, even the therapist. These descriptions are defined as relational episodes (REs), which includes three components: the patient’s expectations or wishes (W) in meeting another person, how the other is considered or expected to react (RO; Response Other) and how the patient responded (RS; Response Self). REs depict real, imagined or dreamt episodes from the patient’s point of view. An RE told by the patient, can be more or less complete. The REs can be collected from therapy sessions or directly from an interview focusing on relationships with central others, as in the Relational Anecdote Paradigm (RAP) [20] (pp 109–120) developed by Crits-Christoph and Luborsky.

In the CCRT method, trained scorers identify and demarcate REs in the transcribed material from therapy sessions or the RAP interview. Other trained independent scorers, not involved in the identification of REs in the transcriptions, identify and categorise W, RO, and RS from the demarcated REs. The most frequent pattern is considered the most useful description of a patient’s CCRT. The goal is to avoid interpreting the patient’s narrative. The chosen categories should reflect literally what the patient told, and inferences should be avoided.

An example of an RE and a tailor-made description of W, RO and RS can illustrate the process. Here is an example of an identified RE:

“He came over, unannounced to have a coffee with me. I pretended to enjoy his visit, since he is a friend since long … but actually I really wanted to sit down and read my book, or rather this was keeping me from reading and that hassled me. I really resented it a lot. With a guy like this, he has helped me a lot before for which I’m grateful. He’s just in his own world … insensitive to others’ needs. And you know he wouldn’t understand if I told him. He would be so sad; you know it was kind of a hassle”. The episode is complete. The tailor-made descriptions can be described as follows: W- to be free of unwanted visitor. RO - “He wouldn’t understand, he would be so sad” and RS - I feel hassled, resentful, guilty and compelled to suffer his presence.

The CCRT standard categories have been empirically chosen from the most frequently used ones, resulting in a standard category list. The third edition is the most widely used. Cluster analysis of the lists of categories resulted in the creation of “clustered standard categories” consisting of eight each for the different components, W, RO and RS [20] (pp. 43–54). From the cited quotation above the following clustered standard categories can be chosen: W: To be distant and avoid conflicts, RO: Upset, RS: Oppose and hurt others (see Table 2).

In an effort to fit in a CCRT pattern into a circumplex model of interpersonal patterns, Crits Christoph et al. [21] developed Quantitative Assessment of Interpersonal Themes (QUAINT). They tried to organise the clusters according to Benjamin’s [22] SASB. A study [23] ended up in 30 W, 31 RO and 40 RS. Most categories of interpersonal behaviour could be rated reliably. However, the study showed poor interrater reliability in some items, mostly among negative wishes.

The original CCRT scoring method is labour intensive and time consuming. First, researchers transcribe therapy sessions or RAP interviews, then independent judges identify REs, and other judges identify the categories in each component and count them to determine the most frequent categories. The categories are ranked according to frequency.

Luborsky and Crits-Christoph’s [11] original work showed an agreement between two raters, scoring 35 cases and reported weighted kappa values ranging from 0.61 to 0.70 in pair comparisons. Since then many studies have been performed with varying levels of reliability. See some examples in Table 1.

**Table 1:** Kappa in CCRT Reliability Studies

Study	Sample	Source of Relational episodes	Reliability
Crits-Christoph et al. (1988) [11]	35 adult patients	Therapy sessions	W: Kw = 0.61 RO: Kw = 0.70 RS: Kw = 0.61
Luborsky et al. (1995) [24]	Mean values in 6 studies	Therapy sessions Reported dreams RAP interviews	W: Kw = 0.63 RO: Kw = 0.66 RS: Kw = 0.69
Zander et al. (1995) [25]	6 nonclinical volunteers	RAP interview	W: K = 0.35 RO: K = 0.41 RS: K = 0.46
Luborsky et al. (2004) [26]	2 patients, 16 scorers, 11 inexperienced	Defined relational episodes	Kw = 0.70 experienced Kw = 0.58 inexperienced
Hegarty et al. (2019) [27]	20 patients with depression	Therapy sessions	W: K = 0.55 RO: K = 0.61 RS: K = 0.57

Notes: *W* Wishes, *RO* Response from Others, *RS* Response from Self. Some results are presented as weighted kappa, others as kappa due to type of scoring in the study. *Kw* Weighted kappa. *K* Kappa [24–27]

Zander et al. [25] scored CCRT directly from a videotaped interview. The results were presented as unweighted kappa, with the following components: W = 0*.35,* RO = 0.*41,* RS = 0*.46;* The study has been criticised by Luborsky and Diguer [24], partly because of its use of kappa instead of weighted kappa which is used in most of the reliability studies on CCRT.

The results indicate that the original CCRT method can be used in research, but it is probably too labour intensive to be used in ordinary clinical practice, supervision or education.

### Aim

The traditional CCRT method has shown itself useful in developing case formulations. Though, it is elaborate and time consuming. The present study aimed to test a simplified method. We investigated rater agreement in a method to establish a CCRT pattern directly from a Dynamic Interview (DI) without transcribing the material.

## Methods

This study is part of the First Experimental Study of Transference-interpretations (FEST). The FEST is a dismantling randomised clinical trial designed to study the long-term effects of transference interpretations in psychodynamic psychotherapy [28].

### Patients

The patients were referred to the study therapists by primary care physicians, private specialist practices and public outpatient departments. No patient was directly recruited for research.

The patients were offered exploratory psychotherapy due to depressive disorders, anxiety disorders, personality disorders and interpersonal problems. Patients with psychosis, bipolar disorder, organic mental disorder or substance abuse were excluded. Written informed consent was obtained from each participant.

After taking history and assessment of background variables by the patient’s therapists, an independent evaluator interviewed each patient in a two-hour psychodynamic semi-structured interview DI, modified after Malan and Sifneos [28]. The patients were asked to talk about themselves and the interviewer should seek to elucidate the dynamics behind the patient’s beliefs, affective experiences, behaviour, maladaptive/adaptive relationships and symptoms. The patients were helped to explore meaningful experiences and vignettes in detail and to give examples of interaction with others in their life. If necessary, they were asked more detailed questions in a manner that illuminated their personal characteristics. When clarifications were used, they were made without elaborations or inferences. In the interview there were no specific focus on the CCRT components.

If patients in about the middle of the interview hadn’t come up with enough information the interviewer asked specifically about parents and friends. The patients were asked to describe their relationships and give concrete examples. Each interview was audiotaped.

### Therapists and evaluators

The patients were assigned to one of seven therapists, depending on the latter’s availability. The therapists comprised six psychiatrists and one clinical psychologist. They had experiences in practising psychodynamic psychotherapy for 10 to 25 years. Four of them were psychoanalysts. The therapists each treated 10 to 17 patients in the study.

### Evaluation and CCRT-scoring

In the FEST the DI [28], was primarily used to obtain the scores on the Psychodynamic Functioning Scales (PFS). The version of the CCRT in this study used the eight standard cluster categories in each of the three components W, RO and RS. The raters scored 58 of the patient interviews The W standard categories used in this study were: To assert self and be independent, To oppose, hurt and control others, To be controlled, hurt, and not responsible, To be distant and avoid conflicts, To be close and accepting, To be loved and understood, To feel good and comfortable, To achieve and help others. The RO categories used were: Strong and independent, Controlling, Upset, Bad, Rejecting and opposing, Helpful, Likes me, Understanding. The RS categories used were: Helpful, Unreceptive, Respected and accepted, Oppose and hurt others, Self-controlled and self-confident, Helpless, Disappointed and depressed, Anxious and shameful.

In all the 58 interviews the information that came forth, was considered sufficient to make CCRT- scoring possible. The scorings were made after listening to the whole interview based on a global impression. One interview was scored by all raters. Most of the interviews were scored by three or four raters (39 patients). The raters were numbered from 1 to 7. The most active raters were number 1 (54 patients), 2 (46 patients), 6 (40 patients), and 7 (34 patients).

In this study more than one category (e.g. controlling and upset) in each component (e.g. response of other) could be chosen, without ranking. This differed from the original method where the frequencies of the categories were registered and ranked according to frequency.. In the FEST, only pairs of raters scoring the same 15 or more patient interviews at a specific time point were included in the analyses. Then the scores presented by the raters were compared in each component (W, RO and RS) respectively.

### Statistics

Cohen’s kappa statistics [29] were used. This method has become the standard for scoring agreement on the CCRT [20] as it takes into consideration agreement occurring by chance.

The data were analysed using SPSS [30] IRR was investigated by comparing every possible combination of pairs of raters, scoring the same patient. Landis and Koch [31], have arbitrarily defined intervals in Cohen’s kappa for inter rater agreement, where 0–0.20 is considered as slight, 0.21–0.40 as fair, 0.41–0.60 as moderate, 0.61–0.80 as substantial, and 0.81–1 as almost perfect agreement.

In the original method raters were instructed to note the frequencies of different categories in each component. When both raters listed the same W, RO or RS as the most frequent, it was given the highest weight. A lower weight was given when one judge’s most frequent score matched the second highest score from the other judge. The lowest weight was given when the two judges’ second highest scores matched. That method made it possible to use weighted kappa.

In this study, kappa was used as there were only categorical data without mutual ranking. The evaluators were never asked to rank the suitability of the chosen categories. Where two raters both scored the same category, it was indicated as 1 and when one or the other scored a category, nor scored by the other it was indicated as 0. Therefore, no weighted kappa could be presented. The kappa was compared between individual raters scoring as in the original study.

## Results

The raters scored one or more categories in each main component (W/RO/RS) without ranking the categories. As shown in Table 1, also in this study, Cohen’s kappa differed among different raters. In total, the results varied from kappa 0.60 to 0.26. The mean values were as follows: W = 0.33 (0.26–0.52), RO = 0.44 (0.29–0.60) and RS = 0.45 (0.31–0.58). CCRT in total: Mean kappa: 0.41. All results were significant.

## Discussion

This study aimed to investigate the reliability in a simplified method of scoring CCRT by calculating IRR among raters’ scoring based on a DI. This less time-consuming method could make CCRT more applicable in psychotherapy research. However, scoring based on the information gathered from an interview used in ordinary clinical practice, makes it difficult to obtain IRR as high as in the original method. Although experienced therapists with considerable training scored the interviews, the results showed an overall fair to moderate IRR (Table 2). DIs were used in this investigation, primarily to score other instruments, but the same interviews were used scoring the CCRT. It was given lesser attention among the raters as it was considered a possible spin off effect of the interview.

**Table 2:** CCRT, inter rater reliability

Rater contra rater	Number of Ratings	Wishes	Response from Others	Response from Self
1 vs 2	27	0.32**	0.44**	0.31**
1 vs 7	18	0.30**	0.53**	0.58**
2 vs 3	16	0.26*	0.48**	0.47**
2 vs 6	20	0.28**	0.60**	0.40**
2 vs 7	19	0.29**	0.29**	0.46**
6 vs 7	15	0.52**	0.32*	0.49**

** *p* < 0.001, * *p* < 0.05

Mean values: Wishes: 0.33 (0.26–0.52) Response from Others: 0.44 (0.29–0.60) Response from Self: 0.45 (0.31–0.58). Mean in total: 0.41. Agreement according to Cohen’s kappa is arbitrary but 0–0.20 is considered as slight, 0.21–0.40 as fair, 0.41–0.60 as moderate, 0.61–0.80 as substantial, and 0.81–1 as almost perfect agreement, referring to Landis and Koch [31].

The ratings on RO and RS showed the highest level of agreement and the W ratings indicated the lowest. These findings, with the least degree of agreement in W, are comparable with those reported by Crits- Christoph and Luborsky [20]. Possibly, this is a sign of raters’ interpreting more, having difficulty to score wishes literally.

In the CCRT method the most frequent category was the one to be chosen, but the second and the third most frequent were also identified, which made weighted data possible [20]. In this study, the raters were instructed to score the observed dominating category (categories), presented in each interview, without any rank. Hence, weighted kappa could not be used.

The raters were asked to score the CCRT from the global impression obtained in the Dynamic interview. Typically, the raters scored four to five categories in total for each patient. The choice to score more than the most dominating, or the most frequent category, was debated by Luborsky and Crits Christoph, who found it more informative to only use the most frequent category in each component [20]. Other studies using CCRT used the opportunity to score more than one category in the different components to declare a more complete description of a patient’s relational patterns [32].

In the studies presented in Table 1, the assessments of IRR vary. Some studies use weighted kappa and some kappa depending on the scoring method. In the present study, there were different numbers of raters scoring the same patient. Additionally, they had the possibility to choose more than one category, without ranking, in each component. This necessarily increased the variance in the results, causing lower kappa values. As noted above, kappa was shown in the present study, whereas weighted kappa is more permissive of varying measures, and therefore as a rule, presents higher kappa values. A previous study showed a weighted kappa for the CCRT pattern in total [26] while the others (see Table 1), showed kappa or weighted kappa for the separate components. In the present study the mean kappa value for the total CCRT pattern resulted in moderate reliability (0.41). Since categorical data were used in this study, its results are not directly comparable with those of Luborsky and Crits Christoph.

A limitation involved the varying numbers of raters scoring the patients. More consistent results might have been obtained with a constant number of raters. Nonetheless, as the raters were compared with one another, and only pairs rating 15 or more patients in common were included in the analyses this limitation presented is a lesser problem.

Scoring more than one item e.g. in W, could increase the chance that one rater is unanimous with another rater in a pair of compared raters, but as Cohen’s kappa is used, if the two raters disagree on another category in the same component, either if one rater just scored one category (and hence disagree) or a different category, it will result in a lower kappa. With ranked categories and the use of weighted kappa, results more similar to other studies might have been obtained.

A strength was that relatively many patients were included in this study compared with other studies investigating IRR in the CCRT. The number of raters in this study could also be considered adequate compared with those in other studies investigating reliability in the CCRT scoring. The raters were experienced clinicians and had participated in earlier research and were skilled in scoring psychometric measures. This might have resulted in higher internal validity but perhaps at the expense of lesser external validity.

The use of kappa as a method to investigate IRR in medical sciences has been criticised, as being far too accepting of low rater agreement considered to be good enough IRR [33]. For example, this issue can be understood when looking at for instance, evaluating a diagnosis of cancer by microscopy, where the raters have to choose “yes” or “no”, as using few categories as a rule results in higher kappa values [34].

In a study performed by Zander and colleagues [25], varying reliability results (0.35–0.46), presented as kappa values, were shown when assessing CCRT directly from videotaped RAP interviews. The present study suggests that at least fair to moderate reliability can be achieved when using a more time-saving procedure, even with interviews primarily used to score other psychometric instruments. The component W as a rule is more difficult to attain reliability in and in this study only reach fair IRR, it might be doubtful to use in research. The other components, RO and RS can possibly be used as they in this study met moderate IRR.

An unanswered question is whether a simplified CCRT scoring based directly on RAP interview, with a focus on REs, without transcribing the interviews, could have improved IRR although this was not the case Zander and colleagues’ study [25] using the German version of the CCRT. In this study a Dynamic interview was used to establish a CCRT pattern. Probably the same interview with more focus on the CCRT and questions to probe for the different components in the CCRT could yield improved agreement. Maybe this simplified scoring method can make it useful in ordinary clinical settings and education.

## Conclusions

This study aimed to investigate IRR in a simplified method of scoring the CCRT based directly on a DI without transcribing the material. The IRR analyses showed fair to moderate rater agreement in a highly controlled research context. Based on this study, RO and RS could be used for research purposes. This study implicates that it is possible to score the CCRT directly from an interview with acceptable IRR.

## Data Availability

The datasets generated and/or analysed during the current study are not publicly available due to privacy but are available from the corresponding author on reasonable request.

## Abbreviations

CCRT: Core Conflictual Relationship Theme
DI: Dynamic interview
FEST: First Experimental Study of Transference–interpretations
IRR: Inter rater reliability
RAP: Relationship anecdotal paradigm
RE: Relational episode
RO: Response from others
RS: Response from self
W: Wishes to others
